# Quack Advertisements

**Published:** 1909-10-30

**Authors:** Eva Richmond


### QUACK ADVERTISEMENTS.

Sir,—I send you an advertisement which seems to merit some attention. It is from a weekly Church paper. I am not a doctor, but I know enough of medicine to realise the harm this sort of advertisement may do. In another form of its advertisement the claim is made that it "expels cancer." How many ignorant people, suffering yet dreading "operation," will be taken in by this, and waste valuable time trying this quack thing? It would be interesting to apply for the "free book" and hunt out some of the "testimonials." Perhaps some reader of your interesting paper may be able to do so. I have noticed the advertisement for some time past, and it seems to me that perhaps I may be incurring the "responsibility of silence" if I say nothing, so I will pass it on to you in the hope that you may be able to deal with it.

Yours faithfully,

Eva Richmond.

[The advertisement enclosed is a particularly offensive one and calculated to mislead.—Ed. The Hospital.]

